# tRNA derived small RNAs—Small players with big roles

**DOI:** 10.3389/fgene.2022.997780

**Published:** 2022-09-19

**Authors:** Suja George, Mohammed Rafi, Maitha Aldarmaki, Mohamed ElSiddig, Mariam Al Nuaimi, Khaled M. A. Amiri

**Affiliations:** ^1^ Khalifa Center for Genetic Engineering and Biotechnology, United Arab Emirates University, Al Ain, United Arab Emirates; ^2^ Department of Biology, College of Science, United Arab Emirates University, Al Ain, United Arab Emirates

**Keywords:** tRNA derived fragments, tRFs, biogenesis, mechanisms of action, recent studies, bioinformatics tools

## Abstract

In the past 2 decades, small non-coding RNAs derived from tRNA (tsRNAs or tRNA derived fragments; tRFs) have emerged as new powerful players in the field of small RNA mediated regulation of gene expression, translation, and epigenetic control. tRFs have been identified from evolutionarily divergent organisms from Archaea, the higher plants, to humans. Recent studies have confirmed their roles in cancers and other metabolic disorders in humans and experimental models. They have been implicated in biotic and abiotic stress responses in plants as well. In this review, we summarize the current knowledge on tRFs including types of tRFs, their biogenesis, and mechanisms of action. The review also highlights recent studies involving differential expression profiling of tRFs and elucidation of specific functions of individual tRFs from various species. We also discuss potential considerations while designing experiments involving tRFs identification and characterization and list the available bioinformatics tools for this purpose.

## Introduction

Transfer RNAs (tRNAs) play a central role in protein synthesis by recognizing the codons in mRNA and recruiting corresponding amino acids for polymerization by the protein synthetase. tRNAs are transcribed by RNA polymerase III and their sizes vary between 73 and 90 nt even though tRNAs as small as 42 nts have been reported (Wende et al., 2014). tRNAs adopt a typical cloverleaf secondary structure consisting of an acceptor stem, D-arm, the anticodon arm, the variable loop, and the T-arm, also known as the TΨC arm. Each arm consists of a double-stranded stem and a single-stranded loop (Cramer et al., 1969; Kim et al., 1974; Robertus et al., 1974; Lyons et al., 2018). However, naturally occurring, active tRNAs that do not fit the established canonical structures have also been described (Dirheimer et al., 1994; Krahn et al., 2020).

tRNAs are encoded by multiple genes in humans, plants, and other organisms (Goodenbour and Pan, 2006; Cognat et al., 2013) and can be present in the nuclear genome and the genome of sub-cellular organelles such as mitochondria and chloroplast (Mohanta et al., 2019; Suzuki et al., 2020). In addition, tRNAs transcribed from the nuclear genome are imported to mitochondria and vice versa (Brubacher-Kauffmann et al., 1999; Tarassov et al., 2007; Rubio and Hopper, 2011). tRNAs are transcribed as precursors (pre-tRNAs, ∼125 nt) which are later processed by ribozymes RNAse P and Z, to cleave the 5′ leader and the 3′ trailer, respectively. After cleavage, a CCA trinucleotide tag is added at the 3′ end of mature tRNAs by a specialized RNA polymerase (tRNA nucleotidyltransferase) (Betat and Mörl, 2015). This addition of CCA trinucleotide is a crucial step in tRNA amino-acylation, export to the cytoplasm, and quality control (Hou, 2010). Several tRNA genes contain introns that are spliced out to generate mature tRNAs (Abelson et al., 1998; Hayne et al., 2020).

Many organisms contain a large number of diverse tRNA genes beyond what is necessary for translation suggesting roles other than protein synthesis (Michaud et al., 2011; Iben and Maraia, 2014). tRNA genes have also been shown to go through rapid evolutionary changes to meet novel translational demands (Yona et al., 2013). In yeast, the loss of a tRNA gene was made up within 200 generations through mutations of another tRNA gene (Yona et al., 2013). tRNA genes are subjected to evolutionary changes in the human population too (Thornlow et al., 2018). Among 1000 human genomes analyzed, over 1% contained 24 new tRNA sequences and over 0.2% of all individuals had 76 new tRNA sequences (Parisien et al., 2013). Considerable evolutionary variations have been observed in organellar tRNAs too (Salinas-Giegé et al., 2015; Zhao et al., 2021). Beyond their role in protein synthesis, additional roles for tRNAs have been reported in the regulation of gene expression, cell wall biosynthesis, post-translational protein labeling, antibiotic biosynthesis, stress responses, priming reverse transcription, and as substrates for non-ribosomal peptide bond formation (Raina and Ibba, 2014).

Even though tRNAs are highly stable compared to mRNAs owing to their highly folded structure and the presence of many post-transcriptional modifications, stringent tRNA surveillance and quality control pathways are in place to prevent the accumulation of nonfunctional tRNAs (Megel et al., 2015). However, several small non-coding RNAs derived from tRNAs have been discovered in species ranging from Archaea to higher plants (Gebetsberger et al., 2012; Xiong et al., 2019; Sun et al., 2022) even though no degradation products are expected to persist after tRNA degradation. Analysis of tRNA defective yeast mutants resulted in a pool of mismodified tRNAs but no significant increase in small non-coding RNAs derived from tRNAs, indicating that these are not a byproduct of random degradation of nonfunctional tRNAs (Thompson et al., 2008). An increasing amount of evidence suggests that these tRNA derived fragments; tRFs, (alternatively known as tRNA derived small RNAs; tsRNAs) are rather specific small non-coding RNAs with distinct sequence structure, specific expression patterns, and biological roles independent of their parental tRNAs (Guan et al., 2020; Chen Q. et al., 2021).

Earlier studies have identified differential expression of tRFs population in various tissues, stresses, and developmental stages and recently, more studies are focusing on functional characterization of individual tRFs. In this review, we summarize our present knowledge on nature, biogenesis, and functions of tRFs and various library construction methods and bioinformatics platforms and tools employed in their identification and characterization.

### Types of tRNA derived fragments (tRFs)

**FIGURE 1:**
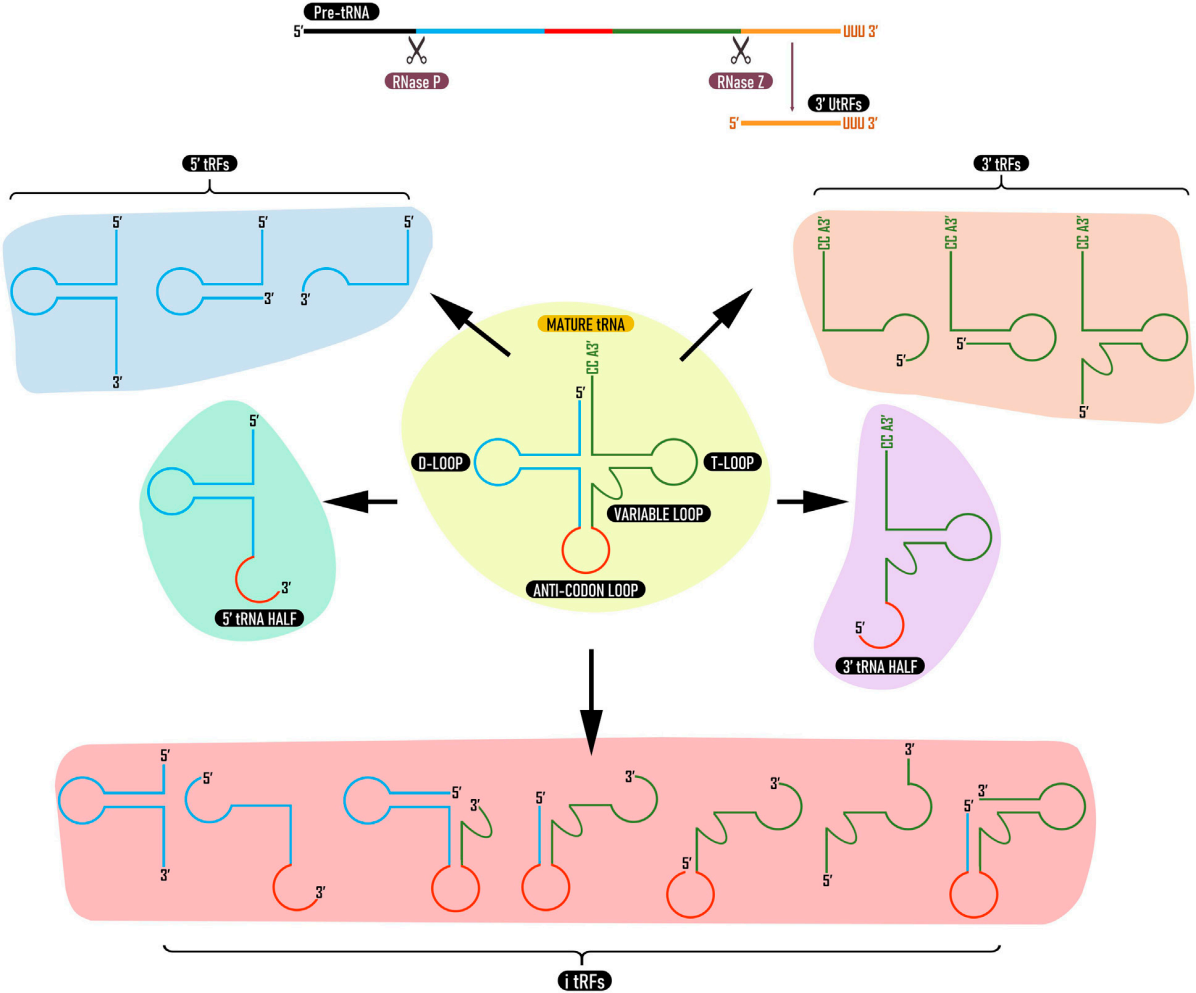
Types of tRNA derived fragments. tRFs can be derived from pre tRNAs (3′ UtRFs) or mature tRNAs (3′ tRFs, 5′ tRFs, i-tRFs, and tRNA halves). 3′ U tRFs are produced by cleavage of pre tRNA by RNase Z (or ELAC2). 3′ tRFs and 5′ tRFs are derived from 3 to 5′ ends of mature tRNA while i-tRFs are derived from internal regions. 3 and 5′ tRNA halves are produced by cleavage at the anticodon loop. tRFs from the same tRNA can vary in type and length depending upon tissue, developmental stage or environmental stimuli.

tRFs are generated either as byproducts of pre-tRNA processing or from mature tRNAs through cleavage by endonucleases. Depending on the location of the cleavage in the tRNA sequence, different types of tRFs are generated (Lee et al., 2009). Later, a classification system for tRFs has been proposed based on their origin and length (Raina and Ibba, 2014); 1) products of cleavage at the 3′ end of the pre-tRNA transcript (3′ U tRFs), 2) products of cleavage at the 3′ end of mature tRNA (3′ CCA tRFs; 3′ tRFs), 3) products of cleavage at the 5′ end of the mature tRNA (5′ tRFs), 4) tRFs that are generated from the interior of the mature tRNA sequence and can include the anticodon (i-tRFs) and 5) tRFs of around ∼35 nt generated by cleavage of mature tRNA molecules in 2 halves (tRNA halves) (Figure 1). The size of tRFs can vary between 10–50 nucleotides (Ma et al., 2021; Yuan et al., 2021). Since then, more comprehensive naming systems have been proposed for tRFs (Megel et al., 2015; Xie et al., 2020; Pereira et al., 2021). Among the five categories, tRFs derived from mature tRNAs are more predominant (Keam and Hutvagner, 2015). In Arabidopsis, more than 99% of tRFs are derived from mature tRNAs and only less than 1% are derived from tRNA precursors (Cognat et al., 2017).

### Biogenesis of tRFs

Different endonucleases have been implicated in the generation of tRFs in different species. *E. coli* prr strain was reported to have a latent tRNA (Lys)-specific anticodon nuclease (prrC) which is released during T4 phage infection. The release of prrC leads to cleavage of host lysine tRNAs at the anticodon loop and helps in restricting phase propagation (Morad et al., 1993). Two other endonucleases from *E. coli*, Colicin E5 and Colicin D also cleave specific tRNAs at the anticodon loop. Colicin D specifically cleaves arginine tRNAs while Colicin E5 cleaves specific tRNAs for Tyr, His, Asn, and Asp (Tomita et al., 2000). In yeast, *Saccharomyces cerevisiae*, Rnase T2 family member Rny1p was found to cleave tRNAs in the anticodon loop (Thompson and Parker, 2009). The heterotrimeric γ-toxin, zymocin, secreted by dairy yeast *Kluyveromyces lactis* is a tRNA endonuclease that cleaves specific tRNA sequences in the anticodon region (Lu et al., 2005). Another tRNA endonuclease that serve as a ribotoxin is PaT from yeast *Pichia acacia* which inhibits growth of *Saccharomyces cerevisiae via* cleavage of tRNA^Gln^ (Chakravarty et al., 2014). Several other ribotoxins reported from microorganisms such as *Mycobacterium tuberculosis*, *Shigella flexneri*, and *Salmonella enterica* are in fact tRNA endonucleases (Winther and Gerdes, 2011; Schifano et al., 2016; Winther et al., 2016).

Several different endonucleases have been reported to generate tRFs in mammalian cells. ELAC2, a homolog of Rnase Z, was found to cleave precursor tRNAs to generate 3′ U tRFs in human prostate cancer cell lines (Lee et al., 2009). In human U2OS cells, ribonuclease angiogenin (ANG), a member of the RNAse A family, cleaves tRNAs to generate tRNA halves in response to arsenite, heat shock, or ultraviolet irradiation (Yamasaki et al., 2009). Recombinant angiogenin induces the production of tRNA halves and inhibits protein synthesis, while Rnase 4 and Rnase A do not show induction of tRNA halves (Yamasaki et al., 2009). Unlike microRNAs which are generated by Dicer, Angiogenin cleaved tRNA halves have the 5′ hydroxyl but not the 5′ phosphate (Lyons et al., 2017). Angiogenin however was found to selectively cleave a subset of tRNAs to produce tRFs in human cell lines (Su et al., 2019). Furthermore, ANG knockout indicated that the majority of stress-induced tRFs are ANG independent. Another recent study of tRNA halves expressed during human renal cell development (Kazimierczyk et al., 2022) observed that in addition to tRFs generated by angiogenin, certain tRFs were cleaved at sites which are different from angiogenin targets. These suggests the existence of other Rnases that produce tRFs in human cells.

Dicer, the central regulator of microRNA processing was investigated as a candidate for generating tRFs. (Cole et al., 2009). showed that generation of 5′ tRFs from tRNA^Gln^ in HeLa cells depended on Dicer. This tRF disappeared after a short siRNA treatment against Dicer confirming that Dicer is required for its generation. Similarly in human renal cells, knockdown of Dicer stopped the biogenesis of 3′ tRFs derived from tRNA^Arg^. The tRF reappeared after transfection of the cells with a plasmid containing the dicer gene further confirming the role of Dicer in its biogenesis (Kazimierczyk et al., 2022). Similarly, Dicer knockdown in human HEK293T cell lines revealed that tRFs and miRNAs were significantly reduced upon Dicer knockdown. tRFs derived from pre-tRNA and tRNA 3′ends were found to be particularly Dicer dependent (Di Fazio et al., 2022). The same study also showed that Dicer specifically binds and cleaves certain tRNAs. Stability of pre-tRNA transcripts may influence cleavage by Dicer (Hasler et al., 2016). It was shown that depletion of Lupus autoantigen La, an RNA-binding protein that stabilizes RNA polymerase III transcripts and supports RNA folding lead to an increase in tRNA cleavage by Dicer in human cells (Hasler et al., 2016).

The biogenesis of tRFs in most plants was found to be mostly independent of Dicer Like proteins (DCLs) (Alves et al., 2017). Expression profiling of tRFs in flower tissues from DCL mutants compared to wild type did not reveal significant variations indicating that DCLs are not primarily essential for the biogenesis of most tRFs in Arabidopsis. Similarly, in rice and other plant species, tRFs biogenesis seemed to be mostly independent of DCLs (Alves et al., 2017). However, the authors did not rule out the possibility that particular components of the miRNA biogenesis machinery might be involved in the biogenesis of particular tRFs. Indeed, the role of DCL1 in the cleavage of specific 19-nt tRFs was later demonstrated in Arabidopsis pollen (Martinez et al., 2017). DCLs may be involved in tRF processing in a tissue/developmental stage/stress-specific manner. S-like Ribonuclease 1 (RNS1), a member of the ancient superfamily of ribonucleases T2/S that is conserved across evolutionarily distant plant species has been shown to generate different tRFs in Arabidopsis (Alves et al., 2017). Interestingly, RNS1 is upregulated under stress conditions indicating a potential role for tRFs in stress regulation (Hillwig et al., 2008).

The extent of posttranscriptional modifications of parent tRNA has been shown to influence the biogenesis of tRFs (Pereira et al., 2021). Queuosine (Q) modification in the wobble anticodon position of some tRNAs was found to inhibit cleavage by endonuclease Angiogenin and alter the pool of tRFs in human cells (Wang et al., 2018). Similarly, methylation by DNA methyltransferase Dnmt2 protected tRNAs against ribonuclease cleavage (Schaefer et al., 2010). tRNA methyltransferase TRMT2A catalyzes the 5-methyluridine (m5U) modification at position 54 of cytosolic tRNAs. M5U54 tRNA hypomodification was observed in TRMT2A mutant human cells followed by an increase in angiogenin-dependent tRFs production. Interestingly, oxidative stress has been shown to downregulate TRMT2A in mammalian cells linking oxidative stress to increased tRFs production (Pereira et al., 2021).

Overall, biogenesis of tRFs is regulated by different endonucleases in different organisms and more than one endonuclease is responsible for tRF biogenesis in many species. More studies and necessary to evaluate the role of different endonucleases in generating subsets of tRFs and their biological implications.

### tRFs are not derived equally from all tRNA genes

Hundreds of tRNA genes are reported in many species to decode the 61 codons (Goodenbour and Pan, 2006). Expression profiles of these tRNAs vary widely, even among isodecoders (tRNAs that share the same anticodon sequence). In human samples, differences in isodecoder tRNA gene expression often did not result in changes in the levels of mature tRNAs but were reflected in the tRFs that are generated from them (Kumar et al., 2014; Torres et al., 2019). In plants too, tRFs were shown to be preferentially derived from a limited set of tRNAs (Cognat et al., 2017; Ma et al., 2021). tRFs derived from different parts of the same tRNA did not show similar abundance, possibly due to differential cleavage of the same tRNA in different regions, differential stabilities of tRFs, or differential selection of tRFs due to their modifications during library construction (Cognat et al., 2017). Different sets of tRNAs may be utilized to generate tRFs in different tissue, stress, and developmental stages.

### Organellar tRFs

tRNAs are transcribed from nuclear, chloroplast, and mitochondrial genomes. It was shown that tRFs generated from the organellar tRNAs could be exported to the cytoplasm and serve as key messengers in nuclear organellar communication (Kumar et al., 2015; Cognat et al., 2017; Meseguer, 2021). tRFs derived from chloroplasts tRNAs (ptRFs) can account for around one-quarter of total short tRFs (19–26 nt) analyzed from Arabidopsis leaves, while tRFs derived from native mitochondrial tRNAs (mtRFs) accounted for >1% of total tRFs. Nuclear and plastid tRNAs displayed rather similar profiles of tRF populations even though they are not expressed in the same compartments (Cognat et al., 2017). Analyzing tRFs originating from mitochondria is more complex. The genomes of several organisms from humans to marsupials contain several copies of their mitochondrial tRNAs in their nuclear genomes (Meseguer, 2021). In addition to tRNAs expressed from true mitochondrial tRNA genes, plant mitochondria contain ‘chloroplast-like’ tRNAs expressed from plastid genes inserted into the mitochondrial genome (Cognat et al., 2017). This is further complicated due to the import of nucleus-encoded tRNAs to mitochondria to compensate for the lack of mitochondrial tRNA genes. Recent studies have been considering these while profiling mitochondrial tRFs (Loher et al., 2017).

### Functional significance of tRFs

**FIGURE 2:**
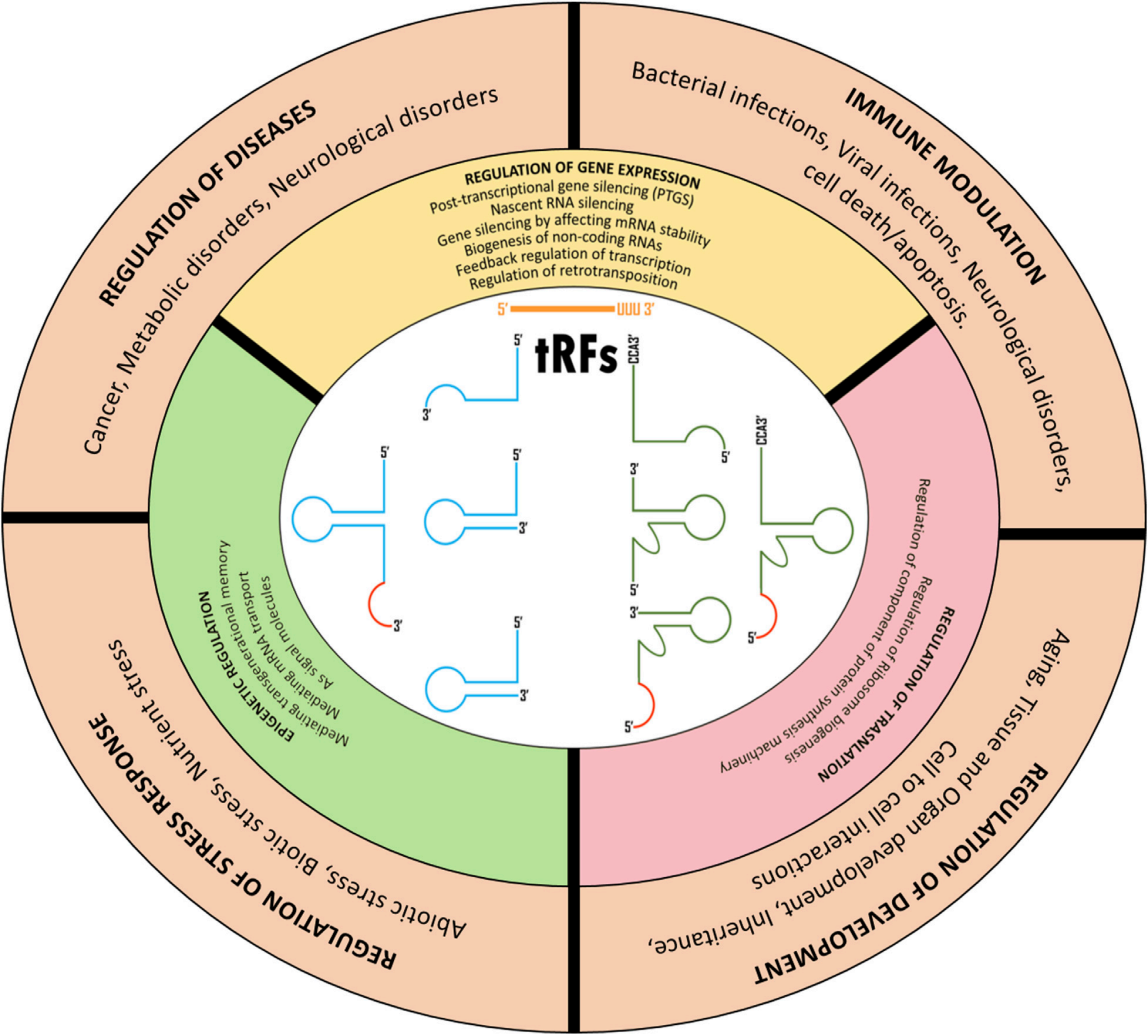
Cellular roles of tRNA derive fragments.

Though the research on tRFs has been gaining momentum only for the past decade or so, several studies have reported their roles in various biological functions. These fall into three main categories; regulation of gene expression, regulation of translation, and epigenetic regulation (Figure 2).

### Regulation of gene expression by tRFs

tRFs can target specific genes and regulate their transcript level through different mechanisms such as Post Transcriptional Gene Silencing (PTGS), nascent RNA silencing (NRS), by affecting mRNA stability, by mediating regulation of retrotransposition and by mediating biogenesis of other non-coding RNAs. Additionally, feedback regulation by specific tRFs to regulate corresponding tRNA gene transcription have also been reported (Rudinger-Thirion et al., 2011; Goodarzi et al., 2015; Schorn et al., 2017; Boskovic et al., 2020; Chen L. et al., 2021; Di Fazio et al., 2022).

The role for tRFs in mediating PTGS have been supported by the discovery of their association with Argonaute proteins (AGOs) (Kuscu et al., 2018; Green et al., 2020). AGOs are key components of the RNA-induced silencing complex (RISC) and play a central role in the regulation of gene expression networks (Kuscu et al., 2018; Müller et al., 2019). tRFs association with AGOs has been shown in a variety of organisms including human, *drosophila* and plants (Loss-Morais et al., 2013; Kumar et al., 2014; Karaiskos et al., 2015; Martinez et al., 2017; Marchais et al., 2019). Through this association similar to miRNAs, the tRF–AGO complex can participate in the RNA silencing and tRFs associated with RISC can induce cleavage of target RNAs (Maute et al., 2013; Kumar et al., 2014; Martinez et al., 2017; Kuscu et al., 2018; Ren et al., 2019). tRFs can bind to their target genes through distinct modes of hybridization (Guan et al., 2020). Some of these binding regions are similar to 5ʹ-localized seed sequences in miRNAs, while others were located at the 3′ end or central region of the tRFs or even extended across the whole tRF molecule. It is possible that tRFs can recognize their targets through multiple binding regions. In human cells, all types of tRFs were found to hybridize with targets even though the most frequent were17–21 nts long (Guan et al., 2020). mRNAs are the main targets of tRFs for PTGS, but a large population of them target various ncRNAs including miRNAs as well (Guan et al., 2020). This, together with previous reports of AGO-loaded miRNAs that can target tRFs (Helwak et al., 2013), suggests that tRFs and miRNAs may regulate each other (Guan et al., 2020).

A novel mode of gene silencing by tRFs, different from transcriptional and post-transcriptional gene silencing was demonstrated in mammalian cells recently (Di Fazio et al., 2022). Dicer generated tRFs were found to guide Ago2-containing silencing complexes to the nucleus to target nascent transcripts for direct cleavage in a sequence specific manner. This nascent RNA silencing occurred through tRFs targeting the initial introns in nascent transcripts, enabling Ago2 to slice them co-transcriptionally. AGO mediated gene silencing inside nucleus by miRNAs are reported in mammalian cells before. But unlike NRS, the gene silencing happened post transcriptionally (Sarshad et al., 2018). It is not clear why miRNA loaded nuclear AGOs do not lead to NRS. (Di Fazio et al., 2022). suggested that unlike miRNAs, the various modifications on tRFs may affect the target RNA:tRF stability contributing to their ability to bind to nascent RNAs. It is highly likely that NRS by tRFs is a key mechanism of gene expression regulation, but more studies in diverse organisms are necessary to confirm this.

A few studies reported that tRFs were able to regulate transcript stability and translation by interacting with RNA Binding Proteins (RBP). YBX1 is an RBP that enhances the translation of several oncogenic transcripts by binding to and stabilizing them (Perner et al., 2022). The majority of YBX1-binding sites in these transcripts were localized to 3′ UTRs and exons (Goodarzi et al., 2015). In breast cancer cells, tRFs derived from specific tRNAs were found to bind to YBX1 in a sequence-specific manner. Competitive binding by these specific tRFs to YBX1 leads to the displacement of oncogenic transcripts resulting in their destabilization, and downregulation. Unlike RNAi-mediated silencing where tRFs serve as guides that direct a transcript to cleavage by endonucleases, here the post-transcriptional silencing is achieved through displacement and destabilization of mRNA transcripts (Goodarzi et al., 2015). In another study, a 32 nt tRF (tRF3E) deriving from the 3′ end of the mature tRNA^Glu^ was shown to specifically bind to NCL (nucleolin), an RBP that binds to several cellular mRNAs, regulating their stability or translation. NCL can interact with a well-known tumor suppressor p53 mRNA and suppress its translation. Competitive binding of tRF3E to NCL leads to increased translation of p53 mRNA (Falconi et al., 2019). A very recent report showed that another tRF, a 5′ tRF from tRNA^Cys^, binds NCL and drives NCL oligomerization to enhance NCL-bound mRNAs’ stabilities. This protected these transcripts from exonucleolytic degradation (Liu et al., 2022). Thus, interactions of specific tRFs with RBPs can lead to increase or decrease in transcript level of associated genes.

tRFs have been reported to regulate reverse transcription of retrotransposons. Mobility of transposable elements (TE) is tightly controlled in cells to prevent mutations. However, epigenetic reprogramming in stem cells release transcriptional control of TEs. Small RNAs are reported to play a major role in inhibiting transposon expression when epigenetic control is compromised by reprogramming (Schorn et al., 2017). Retroviruses, plant pararetroviruses, and retrotransposons require specific tRNAs as primers for reverse transcriptase to initiate DNA synthesis (Marquet et al., 1995). Primer tRNAs bind to complementary primer binding sites (PBS) within the retroelements leading to their reverse transcription. In mouse stem cells, 3′ CCA tRFs have been shown to target PBS in retrotransposons. Competitive binding of tRFs to PBS prevents their reverse transcription and retrotransposition. Thus, the presence of specific tRFs could provide an innate immunity during horizontal entry of LTR-retrotransposons (Schorn et al., 2017).

Specific tRFs have been shown to play a role in the production of a wide variety of other noncoding RNAs such as snoRNAs, scaRNAs, and snRNAs (Boskovic et al., 2020). Cajal bodies are coiled suborganelles present in the nucleus involved in ribonucleoprotein processing and maturation. A 5′ tRF from tRNA^Gly^ was found to positively regulate production of non-coding RNAs such as U7 snRNA in Cajal bodies of human and mouse embryonic stem cells. In addition, tRFs regulate the biogenesis of Cajal bodies and in turn regulate the biogenesis of other non-coding RNAs (Boskovic et al., 2020).

In addition to regulating transcript level of other genes and noncoding RNAs, tRFs are known to regulate the transcript level of their parent tRNA (Chen L. et al., 2021). In zebrafish embryos, 5′ tRFs from tRNA^Glu^ and tRNA^Gly^ were found to promote transcription of matching tRNA genes. Knockdown of 5′ tRFs resulted in significant reduction in expression levels of corresponding parent tRNA while synthetic 5′ tRFs mimetics that do not possess native modifications were able to significantly increase the parent tRNA levels. This feedback regulation was caused by binding of 5′ tRFs to the template strand of their corresponding tRNAs. This binding of 5′ tRFs to the template strand prevent homologous full-length tRNAs from forming transcriptionally inhibitory RNA:DNA hybrids on the same tRNA genes, promoting their transcription (Chen L. et al., 2021).

### Regulation of translation by tRFs

tRFs have been shown to regulate translation of various proteins through participating in ribosome biogenesis as well as directly interacting with various components of protein synthesis machinery.

A role for tRFs in fine-tuning the production of ribosomal proteins and ultimately the number of ribosomes in proliferating cells was demonstrated in human cancer cells (Kim et al., 2017). A 3′ tRF from tRNA^LeuCAG^ was shown to enhance translation of small ribosomal subunit protein mRNA, RPS28 (Kim et al., 2017). The authors proposed that this tRF is involved in unfolding the mRNA target sites that have duplexed secondary structures allowing for enhanced translation. The tRF target site in the RPS28 coding sequence is conserved in vertebrates indicating that tRF-regulated mRNA translation of RPS28 is a conserved process (Kim et al., 2019). RPS28 is needed for ribosomal RNA 18S rRNA biogenesis and is an integral part of the 40S ribosomal subunit (Robledo et al., 2008). Inhibition of this tRF induces apoptosis in rapidly dividing cells by impairing ribosome biogenesis (Kim et al., 2017).

Direct interaction of tRFs with ribosomes and inhibition of global translation was shown in *Haloferax volcanii* (Gebetsberger et al., 2012). In this halophilic archaeon, a 5′tRF processed in a stress-dependent manner from valine tRNA was found to primarily target the small ribosomal subunit leading to reduction in protein synthesis by interfering with peptidyl transferase activity (Gebetsberger et al., 2012). In yeast too, specific tRFs were found to associate with small ribosomal subunits to inhibit translation by affecting global aminoacylation and the aminoacylation of the corresponding parental tRNAs (Mleczko et al., 2018). In mammalian cells, a 5ʹ tRNA half derived from tRNA^Pro^ was found to be associated with ribosomes and polysomes (Gonskikh et al., 2020). Addition of synthetic tRNA^Pro^ halves to mammalian *in vitro* translation systems results in global translation inhibition. The authors suggested that binding of the tRNA^Pro^ 5ʹ half to the ribosome leads to ribosome stalling resulting in translation inhibition (Gonskikh et al., 2020).

Significant inhibition of global protein synthesis by tRFs through interaction with specific components of translation machinery was demonstrated in human cells (Yamasaki et al., 2009). This inhibition of translation by angiogenin cleaved tRFs under stress conditions is associated with increased accumulation of stress granules too (Ivanov et al., 2011). Later studies showed that this inhibition of translation involves 5′ tRFs derived from tRNA^Ala^ and tRNA^Cys^ and correlates with their ability to displace eIF4G/A from mRNA. These tRFs interact with the translation repressor Y-box-binding protein 1 (YB-1) and displace the cap-binding complex to prevent eIF4G/A from initiating translation. (Ivanov et al., 2011). Another 5′ tRF, derived from tRNA^Gln^ (Gln19) was found to modulate translation by associating primarily with the human multisynthetase complex (MSC) (Keam et al., 2017). In model plant Arabidopsis, translation inhibition by tRFs possibly through association with active polyribosomes has been reported (Zhang et al., 2009; Lalande et al., 2020). In Drosophila, tRFs regulate the global translational activities by targeting key components of translation machinery through conserved antisense sequence matching in an AGO-dependent manner largely independent of miRNA-mediated regulation (Luo et al., 2018).

While most studies on tRF mediated regulation of translation reported inhibition of translation, a role for tRFs in stimulating translation was demonstrated in the pathogenic parasite *Trypanosoma brucei*. tRFs derived from tRNA^Thr^ accumulated during nutrient deprivation and enhanced translation by facilitating mRNA loading on ribosomes and polysomes (Fricker et al., 2019).

### Epigenetic regulation of gene expression by tRFs

Epigenetic changes are dynamic and heritable genetic alterations without any nucleotide changes (Park et al., 2020). tRFs have been reported to function in epigenetic regulation by acting as signal molecules, influencing mRNA transport and affecting transgenerational memory.

Rhizobial tRFs have been shown to act as signal molecules that positively regulates host nodulation (Ren et al., 2019). The rhizobium *Bradyrhizobium japonicum* was found to produce tRFs derived from all 50 rhizobial tRNAs in both Rhizobium culture and nodules of the host plant soybean. The tRFs were more abundant in nodules than in culture and were primarily 3′ tRFs. These tRFs were predicted to target 52 soybean genes but were not predicted to target rhizobial genes. Three rhizobial tRFs, derived from tRNAs, Val-1-tRNA (CAC), Gly-1-tRNA (UCC), and Gln-1- tRNA (CUG) were to regulate host genes that were negative regulators of nodulation through cleavage of mRNAs in predicted target sites in a manner similar to miRNAs. Interestingly, none of the predicted target sites for rhizobial tRFs were not predicted to be targeted by previously identified soybean small RNAs (Arikit et al., 2014; Ren et al., 2019). The rhizobial tRFs were shown to utilize the soybean AGO1 to catalyze tRF-guided cleavage of target mRNAs in the host cells (Ren et al., 2019). These results indicate a cross-kingdom communication involving tRFs and their targets. It is possible that such cross-kingdom communication involving tRFs exist between other host-symbiont/parasite relationships too. Additionally, such communication could exist between disease causing agents and their hosts. Further studies in different species are necessary to evaluate such roles of tRFs.

A role for tRFs in movement of mRNAs graft junction has been observed in grafted plants of different Arabidopsis ecotypes (Thieme et al., 2015). Plants use the movement of macromolecules through phloem as a mechanism for long-distance signaling to affect distal regions (Mach, 2016). The abundance of tRNAs in phloem sap correlated with the movement of a large number of mRNAs across graft junction suggesting a potential role for them in mRNA mobility (Thieme et al., 2015). tRNAs can interact with the 3′ ends of mRNAs to create a linked RNA that can move through plasmodesmata (Westwood and Kim, 2017). Synthetically constructed tRFs derived from different regions of tRNA^Met^ could facilitate systemic movement of co-transcribed mRNA between roots and shoots in transgenic systems while retaining the mRNA function (Zhang et al., 2016). Additional studies are required to verify if naturally occurring tRFs associate with mRNAs to influence their movement.

Importance of tRFs in transgenerational memory has been demonstrated in widely unrelated species. Sperm tRFs from paternal mice on a high-fat diet altered gene expression of metabolic pathways in early embryos and islets of F1 offspring leading to metabolic disorders (Chen et al., 2016). Similarly, sperm tRFs from aged male mice induced anxiety-like behaviors in F1 males (Guo et al., 2021) and mediated obesogenic and hedonic phenotypes in the progeny following maternal high-fat diet (Sarker et al., 2019). Another interesting study showed that sperm cells from nutrient restricted mice gained tRFs from tissue in the reproductive tracts through fusion with tRFs containing vesicles. These tRFs were passed on to the embryos where they were found to repress genes associated with the endogenous retroelement MERVL (Sharma et al., 2016). Post-transcriptional modifications of tRFs are potentially important in intergenerational inheritance of metabolic disorders as deletion of tRNA methyltransferase, DNMT2, abolished sperm sncRNA-mediated transmission of high-fat diet-induced metabolic disorders to offspring (Zhang et al., 2018). In *Brassica rapa,* changes in expression profiles of tRFs in heat-stressed plants were inherited by unstressed progeny leading to improved thermotolerance compared to unprimed plants (Byeon et al., 2019). It has been proposed that small RNAs including tRFs could serve as signaling molecules that transmit between tissues and even across generations to bring out transgenerational memory (Zhang and Tian, 2022).

### Identification and expression profiling of tRFs

Several studies have reported differential expression of tRFs under various conditions in many species. Population and expression levels of tRFs were found to vary between cancerous and normal tissues in mouse and human cells indicating potential roles in carcinogenesis (Jehn et al., 2020; Huang et al., 2022). Differential expression of tRFs is reported across various tissues and developmental stages (Soares et al., 2015). In plants, differential expression of tRFs is reported across various tissues, between developmental stages, and under abiotic and abiotic stresses (Cognat et al., 2017; Meijer et al., 2022; Sun et al., 2022). Table 1 lists recent studies reporting expression profiling of tRFs from various species.

**TABLE 1:** Differential Expression profiling of tRFs in various organisms.

Sl. No.	Organism	Tissue/Stress/Developmental stage	References
**Human cells**			
1	Human	clinical lung adenocarcinoma tissues and adjacent normal lung tissues	Huang et al. (2022)
2	Human	primary nasopharyngeal carcinoma tissues and healthy controls	Lu et al. (2021)
3	Human	high-grade serous ovarian cancer (HGSOC) and adjacent normal ovarian tissues	Chen et al. (2021a)
4	Human	Aorta tissue from Aortic dissection (AD) patients and healthy controls	Fu et al. (2021)
5	Human	Peripheral blood mononuclear cells (PBMCs) from blood samples of IgA nephropathy (IgAN) patients and healthy control groups	Luo et al. (2020)
6	Human	plasma cells from the bone marrow of diagnosed myeloma and healthy donors	Xu and Fu, (2021)
7	Human	bone marrow stromal cells (BMSCs) from Fibrous dysplasia (FD) patients and healthy controls	Ling et al. (2022)
8	Human	clinical breast cancer tissues and adjacent normal samples	Wang et al. (2019)
9	mouse, rat, pig, human, chimpanzee, macaque	Hippocampal tissue	Jehn et al. (2020)
10	Human	Muscle-invasive bladder cancer (MIBC) specimens and adjacent control mucosal tissues	Qin et al. (2022)
**Plants**			
1	Arabidopsis	Phosphate sufficient and deficient shoot and root samples	Hsieh et al. (2009)
2	Arabidopsis	Various tissues and stresses	Cognat et al. (2017)
3	Arabidopsis	Shoot tissue from cold treated and control plants	Tiwari et al. (2020)
4	Arabidopsis, rice	Various tissues, stress, and developmental stages	Ma et al. (2021)
5	Rice (*Oryza sativa*)	Various tissues and developmental stages	Meijer et al. (2022)
6	Tomato (*Solanum lycopersicum*)	exogenous abscisic acid (ABA) treated leaf tissue and control untreated leaves	Luan et al. (2020)
7	Wheat (*Triticum aestivum*)	Leaves from heat-treated seedlings	Wang et al. (2016)
8	Wheat (*Triticum aestivum*)	spikelets inoculated with Fusarium graminearum and uninoculated controls	Sun et al. (2022)
9	Barley (*Hordeum vulgare* L.)	Phosphate sufficient and deficient shoot and root samples	Hackenberg et al. (2013)
10	Barley (*Hordeum vulgare* L.)	Phosphate sufficient and deficient shoot and root samples	Sega et al. (2021)
11	Black Pepper (*Piper nigrum* L.)	*Phytophthora capsica* infected leaves and roots	Asha and Soniya, (2016)
12	*Brassica rapa*	Various tissues	Byeon et al. (2019)
13	Different land plants	Various tissues	Alves et al. (2017)
**Others**			
1	*Drosophila melanogaster*	Various tissues and developmental stages	Luo et al. (2018)
2	*Drosophila melanogaster*	Ovary tissues from wild type and tRNA biogenesis mutants	Molla-Herman et al. (2020)
3	Mice	Sperm cells from mice exposed to cadmium	Zeng et al. (2021)
4	Mice	Retina from mice with oxygen-induced retinopathy (OIR) and control mice	Peng et al. (2020)
5	Mice	Brain tissue from SAMP8 (senescence-accelerated mouse prone 8) mice and SAMR1 (senescence-accelerated mouse resistant 1) mice	Zhang et al. (2019)
6	mice	Skin tissue of ultraviolet Irradiated and untreated animals	Fang et al. (2021)
7	*Schizosaccharomyces pombe*	Heat stressed and unstressed samples	Hu et al. (2021)
8	*Schmidtea mediterranea*	Transverse sections of the organism	Lakshmanan et al. (2021)
9	*Cryptococcus gattii, Cryptococcus neoformans*	Comparison between the two basidiomycetous yeasts	Streit et al. (2021)
10	Zebrafish (*Danio rerio*)	Various tissues and developmental stages	Soares et al. (2015)

### Functional analysis of specific tRFs

Research interest in tRFs is rapidly gaining momentum, and many studies are reporting differential expression of tRFs in the experimental conditions of interest (Table 1). tRFs have been reported to play major roles in many cancers (Li X. et al., 2021). (Telonis et al., 2019) carried out a large-scale investigation of tRF-mRNA co-expression networks in 32 cancer types. Their results showed that mitochondria contribute disproportionally more tRFs than the nuclear ones. The associations between specific tRFs and mRNAs were found to differ from cancer to cancer. Correlation of tRFs with mRNAs depended on their length and presence of higher density in repeats, such as ALUs, MIRs, and ERVLs. Detailed analysis of tRF-mRNA cancer specific associations would help in identifying candidate genetic elements for targeted treatments.

The studies involving functional characterization of individual tRFs are limited mostly to cancer research. Some tRFs were reported to promote cancer progression while others were reported to suppress it. A 3′tRF from tRNA^Val^ was significantly upregulated in gastric cancer tissues compared to control tissues and was positively correlated with tumor size and the depth of tumor invasion (Cui et al., 2022). Further functional analysis proved its involvement in cancer proliferation and invasion and inhibition of apoptosis in gastric cancer cells *via* binding to chaperone molecule EEF1A1 (Cui et al., 2022). Another study showed that tRF-3017A (derived from the 3′ end of mature tRNA^Val^) was involved in silencing tumor suppressor NELL2 leading to significantly higher lymph node metastasis in gastric cancer (Tong et al., 2021). Interestingly, another tRNA^Val^ derived tRF, tRF-19-3L7L73JD, was shown to have a potential role in the suppression of the development of gastric cancer (Shen et al., 2021). tRF-19-3L7L73JD was downregulated in plasma from gastric cancer patients compared to healthy controls. It was found to inhibit cell proliferation and migration, induced apoptosis, and arrested cells at G0/G1 phases (Shen et al., 2021). The expression level of a 5′ tRF from tRNA^Arg^ (tRF-20-S998LO9D), was positively correlated with poor prognosis in a variety of cancers such as breast invasive carcinoma, head and neck squamous cell carcinoma, kidney renal clear cell carcinoma, lung squamous cell carcinoma, pheochromocytoma and paraganglioma, and uterine corpus endometrial carcinoma (Gu et al., 2020; Ma and Liu, 2022). Specific tRFs have been suggested as predictive biomarkers and intervention targets for several types of cancers (Sun et al., 2018; Xi et al., 2021).

Although there are several published studies on differential expression of tRFs under various stress conditions, tissues and developmental stages in many plant species, (Table 1), functional analysis of specific tRFs are limited. In a recent report, a 5′ tRF from tRNA^Ala^ was found to negatively regulate Cytochrome P450 71A13 (CYP71A13) expression and camalexin biosynthesis in Arabidopsis *via* AGO1 mediated silencing. Negative regulation of Camalexin, the major phytoalexin in Arabidopsis, lead to decreased resistance to fungal pathogen *Botrytis cinerea* (Gu et al., 2022). Table 2 lists a few of the reported functional studies in tRFs.

**TABLE 2:** Documented roles of specific tRFs.

Sl. No.	Type of tRF/name	Parental tRNA	System	Function	References
1	3′ tRF	Leu^CAG^	Human cells	Enhances translation of specific ribosomal protein mRNAs, regulates ribosome biogenesis	Kim et al. (2017)
2	-	tRNA^Glu^, tRNA^Asp^, tRNA^Gly^, tRNA^Tyr^	Human cells	Suppress Breast Cancer Progression	Goodarzi et al. (2015)
3	3′ tRF (tRF3008A)	tRNA^Val^	Human cells	Suppresses the progression and metastasis of colorectal cancer	Han et al. (2022)
4	3′ tRF (tRF-3019a)	tRNA^Ala^	Human cells	Enhances cell proliferation, migration, and invasion in gastric cancer	Zhang et al. (2020a)
5	5′ tRF	tRNA ^Lys^	Human cells	Early Progression of bladder cancer	Papadimitriou et al. (2020)
6	3′ tRF	tRNA^GIu^	Human cells	Tumor suppressor in breast cancer	Falconi et al. (2019)
7	5′ tRF	tRNA^Gly^	Human cells	Controls noncoding RNA production and histone levels	Boskovic et al. (2020)
8	5′ tRNA half	tRNA^His^	Human cells	Functions in the innate immune response by activating Toll-like receptor 7	Pawar et al. (2020)
9	5′ tRNA half	tRNA^His^	Human cells	Functions in B-lymphoblastic Cell Proliferation	Mo et al. (2020)
10	3′ tRNA half (tsRNA-16902)	tRNA^Thr^	Human cells	Regulates the adipogenic differentiation of human bone marrow mesenchymal stem cells	Wang et al. (2020)
11	5′ tRF	tRNA^Gln^	Human cells	Promotes Respiratory Syncytial Virus replication and induction	Choi et al. (2020)
12	5′ tRF	tRNA^Glu^	Human cells	Regulates Breast Cancer Anti-Estrogen Resistance 3 (BCAR3) expression and proliferation in ovarian cancer cells	Zhou et al. (2017)
13	5′ tRNA halve	tRNA^Gly^	Human cells	Directly binds to splicing-related RNA-binding protein RBM17 and regulates papillary thyroid cancer	Han et al. (2021)
14	tRF3	tRNA^Thr^	Human cells	Target the 3′UTR of Z-DNA-binding protein 1 (ZBP1) for its degradation leading to the suppression of acute pancreatitis	Sun et al. (2021)
15	5′ tRF (TRF365)	tRNA^Thr^	Human cells	Regulates the metabolism of anterior cruciate ligament (ACL) cells by silencing the expression of The inhibitor of nuclear factor kappa B kinase subunit beta	Long et al. (2022)
16	5′ tRF	tRNA^Ala^	Arabidopsis	Repress anti-fungal defense by negatively regulating Cytochrome P450 71A13 (CYP71A13) expression in an AGO1dependent manner	Gu et al. (2022)
17	5′ tRF	tRNA^Gln^	Porcine male germ cells	Regulates early cleavage of preimplantation embryos in mature spermatozoa	Chen et al. (2020)

### Identification, expression profiling, and functional analysis of tRFs–Methods and considerations

Next-generation sequencing technologies and microarrays are effective tools for high throughput identification and expression profiling of tRFs. tRNAs are heavily modified post-transcriptionally (Lyons et al., 2018; Delaunay and Frye, 2019). More than 170 RNA modifications are reported and over 90 such modifications are found in tRNA, even though their frequency and distribution vary according to organism or tRNA species (Lyons et al., 2018). These post-transcriptional modifications serve to stabilize the tertiary structure, are required for efficient export to cytoplasm, and aid in the codon-anticodon recognition and translation (Lipowsky et al., 1999; Bednářová et al., 2017). Modifications including mononucleotides that did not correspond to the four canonical RNA bases such as pseudouridine (Ψ), 2-methyladenine, N6-methyladenine, N6-dimethyladenine, etc, and 2′-O-methylation are common in tRNAs from various species (Hou et al., 2015; Bednářová et al., 2017; Lyons et al., 2018). tRNA modifications influence the specificity and efficiency of tRNA cleavage to produce tRFs (Schaefer et al., 2010; Wang et al., 2018). Similar to tRNAs, tRFs are also rich in post-transcriptional modifications which could potentially play roles in their function and should be considered in studies involving the identification and characterization of tRFs (Guzzi et al., 2018; Akiyama et al., 2020). Post-transcriptional modifications in tRFs often lead to misincorporation by reverse transcriptase and interfere with the efficiency of reverse transcription (Ebhardt et al., 2009; Potapov et al., 2018) and ultimately affect the tRFs population identified through small RNAseq library preparation. Some tRFs contain a 2′,3′-cyclic phosphate (cP) at their 3′ termini that inhibit the adapter ligation reaction (Honda et al., 2016). Several newly developed sequencing techniques such as ARM-seq, cp-RNA-seq, hydro-tRNAseq, and YAMATseq have taken these into consideration (Cozen et al., 2015; Honda et al., 2016; Gogakos et al., 2017; Shigematsu et al., 2017). These techniques have incorporated RNA pretreatments such as demethylation, selective amplification of RNA fragments that contain 3′-cP, partial alkaline RNA hydrolysis, 3′-terminal diacylation, 3′-cP removal, and 5′-phosphate addition, use of specific adapters for ligation, etc. (Ma et al., 2021).

Additionally, the size of tRFs in a population can vary, with tRNA halves ranging from 30 to 40 nt, tRFs derived from D and T arms ranging from 18 to 25 nt, and internal tRFs as small as 10 nt (Alves and Nogueira, 2021; Ma et al., 2021; Yuan et al., 2021). Hence, studies involving tRFs identification should consider this while doing size selection.

### Bioinformatics analysis of tRFs

Mapping the tRFs to tRNA space alone, instead of the full genome can lead to the generation of false positives (Telonis et al., 2016). This is because well-studied genomes like the human genome contain hundreds of incomplete tRNA sequences which do not produce functional tRNAs. Mapping pipelines that do not map to the full genome might erroneously map these deep-sequencing reads as tRFs because of the existing sequence similarity (Telonis et al., 2016). 3′tRFs contain the non-templated “CCA” at the 3′ terminus, which can also exist at multiple genomic locations that are unrelated to tRNAs, and it is essential to rule out them to eliminate false positives (Telonis et al., 2016). Mapping the small RNA reads to both tRNAs and the full genome and discarding the reads that map better to non-tRNA space would help to eliminate false positives (Thompson et al., 2018; Ma et al., 2021).

Post-transcriptional modifications affect RNA polymerase and reverse transcriptase fidelity (Potapov et al., 2018) and this needs to be considered while permitting mismatches when mapping the reads sequences on the mature tRNA sequences (Cognat et al., 2017). It was shown that permitting two mismatches did not lead to an inflated number of false-positive tRFs in Arabidopsis (Cognat et al., 2017). A post-transcriptional modification of “m1A” at position 58 is highly present in Arabidopsis tRNAs and often leads to misincorporation by reverse transcriptase (Chen et al., 2010; Cognat et al., 2017). Permitting one mismatch was shown to be essential to accurately retrieve the set of 3′ tRFs with this modification (Cognat et al., 2017).

### Identification of tRF interacting proteins and RNAs

Techniques such as CLIP (Crosslinking and Immunoprecipitation), PAR-CLIP (Photoactivatable Ribonucleoside-Enhanced Crosslinking and Immunoprecipitation), and CLASH (crosslinking, ligation, and sequencing of hybrids) are used widely for identifying tRFs interacting with AGO proteins and other RNAs (Kudla et al., 2011; Spitzer et al., 2014; Hafner et al., 2021). PAR-CLIP incorporates photoreactive ribonucleoside analogs into nascent transcripts. Upon UV exposure, photoreactive nucleoside-labeled cellular RNAs are cross-linked effectively to their interacting RBPs. After immunoprecipitating and purifying the Cross-linked RNA-RBP complexes, protein is digested and the RNA is reverse transcribed and sequenced. Binding sites of the proteins on the RNA can be recognized by mutations caused by incorporated ribonucleoside analogs (Danan et al., 2016). CLASH identifies RNA-RNA interactions. Similar to PAR-CLIP, protein associated RNAs are stabilized by cross-linking the RNAs with proteins by ultraviolet irradiation and the proteins are immunoprecipitated. RNAs associated with proteins are partially truncated and a ligation step physically connect the ends of RNA-duplexes in an individual protein molecule. Later, these RNAs are reverse transcribed. The ligated duplexes give rise to chimeric cDNAs, enabling the identification of RNA-RNA interaction sites (Helwak and Tollervey, 2014).

PAR-CLIP has been useful in deciphering specific AGO associations of tRFs. Human PAR-CLIP data showed that 5′ and 3′ tRFs preferentially associated with AGO1, 3 and 4 rather than AGO2 (Kumar et al., 2014). Dicer PAR-CLIP data from human HEK293T cell line suggested that Dicer is involved in the biogenesis of a subset of tsRNAs (Di Fazio et al., 2022). Human AGO1 CLASH identified thousands of RNAs that interact with tRFs (Kumar et al., 2014).

### Functional characterization of tRFs

Owing to the potential roles post-transcriptional modifications play in tRFs function (Guzzi et al., 2018), the use of synthetic RNAs that closely mimic endogenous tRFs in their sequence may not be ideal for functional studies (Akiyama et al., 2020). Purification of tRFs retaining modifications from endogenous sources and their use in functional studies would be more prudential in this context. In a recent study, the authors used DNA oligo probes complementary to target tRFs to efficiently isolate natural individual tRFs containing internal modifications (Akiyama et al., 2020). The translation inhibition efficiency of thus isolated endogenous tRF (5ʹ-tiRNA^Gly^) was greater than its synthetic counterpart (Akiyama et al., 2020). Different *in vitro* and *in vivo* strategies for the production of tRFs retaining modifications have been described here (Drino et al., 2020). For knockdown studies involving tRFs, Short tandem target mimic (STTM) technology can be useful (Tang et al., 2012). STTM is a synthetic, short, non-coding RNA that can block the function of small RNAs of interest. STTM has been successfully used knockdown specific tRFs and decipher their function (Martinez et al., 2017). It would be prudent to consider the evolutionary variability of tRNA genes (Parisien et al., 2013) and in turn tRFs even within the population of single species while designing functional characterization experiments.

### Bioinformatics resources for tRNAs and tRFs

Several databases and bioinformatics tools exist for studies focusing on tRNA and tRFs. tRFdb is a database that contains tRFs from 8 species including humans and mice (Kumar et al., 2014). PtRFdb is a database for plant transfer RNA-derived fragments (Gupta et al., 2018). It includes information on tRFs from ten different plant species. The database also includes sequence similarity searches. Table 3 lists available databases and tools for tRNAs and tRFs.

**TABLE 3:** Databases and tools for tRNAs and tRFs.

Name	Details	Website	Ref
**tRNA databases and tools**			
GtRNAdb 2.0	Database of tRNA genes from over 4370 genomes, includes information on tRNA modifications, SNPs, gene expression, and evolutionary conservation	http://gtrnadb.ucsc.edu/	Chan and Lowe, (2016)
DBtRend	Database of mature tRNA expression profiles across various biological conditions in humans	https://trend.pmrc.re.kr/	Lee et al. (2021)
PLMItRNA	Database of plant mitochondrial tRNAs and tRNA genes	http://bio-www.ba.cnr.it:8000/srs/	Ceci et al. (1999)
tRNAscan-SE	Tool for predicting tRNA genes in whole genomes	http://trna.ucsc.edu/tRNAscan-SE/	Chan and Lowe, (2019)
tRNAmodpred	Tool for prediction of posttranscriptional modifications in tRNAs	http://genesilico.pl/trnamodpred/	Machnicka et al. (2016)
PRMdb	Includes predicted tRNA modifications in plants	http://www.biosequencing.cn/PRMdb/	Ma et al. (2020)
tRic	Database of tRNA expression profiles in 31 human cancer types	https://hanlab.uth.edu/tRic	Zhang et al. (2020b)
**tRFs databases and tools**			
tRFdb	Database of tRFs from 8 species including human, mouse, *drosophila*	http://genome.bioch.virginia.edu/trfdb/	Kumar et al. (2015)
PtRFdb	Database of tRFs from plant species including Arabidopsis, rice, soybean, sorghum, maize, etc	www.nipgr.res.in/PtRFdb	Gupta et al. (2018)
tRFexplorer	Expression profiles of tRFs in TCGA and NCI-60 panel cell lines (human tumor cell lines)	https://bio.tools/tRFexplorer	La Ferlita et al. (2019)
MINTbase	Database of nuclear and mitochondrial tRFs from all The Cancer Genome Atlas projects	https://cm.jefferson.edu/MINTbase/	Pliatsika et al. (2018)
MINTmap	Tool for identification of mitochondrial and nuclear tRFs in short RNA-seq datasets	https://github.com/TJU-CMC-Org/MINTmap/	Loher et al. (2017)
tRFtarget	Database of predicted tRFs targets in eight species	http://trftarget.net	Li et al. (2021a)
tRFTars	A tool for predicting the targets of tRNA-derived fragments	http://trftars.cmuzhenninglab.org:3838/tar/	Xiao et al. (2021)
tRFTar	Tool for prediction of tRF-target gene interactions	http://www.rnanut.net/tRFTar/	Zhou et al. (2021)
miRge3.0	tRF sequencing analysis pipeline	https://anaconda.org/bioconda/mirge3	Patil and Halushka, (2021)
tDRmapper	A tool for mapping, naming and quantifying tRFs	https://github.com/sararselitsky/tDRmapper	Selitsky and Sethupathy, (2015)
SPORTS1.0	A tool that includes annotation and profiling of tRFs	https://github.com/junchaoshi/sports1.0	Shi et al. (2018)
tsRNAsearch	A pipeline for identification of differentially expressing tRFs from small RNA-sequencing data	https://github.com/GiantSpaceRobot/tsRNAsearch	Donovan et al. (2021)
deepBase v3.0	tRFs annotation and expression profiles in various cell lines	http://rna.sysu.edu.cn/deepbase3/index.html	Xie et al. (2021)
tRex	Database of tRFs in Arabidopsis	http://combio.pl/trex	Thompson et al. (2018)
tsRFun	Database and tools for tRF analysis across 32 types of cancers	http://rna.sysu.edu.cn/tsRFun/	Wang et al. (2022)
OncotRF	Database and tools for exploring tRFs in human cancers	http://bioinformatics.zju.edu.cn/OncotRF	Yao et al. (2020)
tRF2Cancer	Database and tools to analyze expression profiles of tRFs in multiple cancers	http://rna.sysu.edu.cn/tRFfinder/	Zheng et al. (2016)
tRFanalyzer	Database of tRNA and tRFs expression data from Arabidopsis and rice	http://www.biosequencing.cn/tRFanalyzer/	Ma et al. (2021)
tsRBase	Database for expression and function of tRFs in many species	http://www.tsrbase.org	Zuo et al. (2021)

## Conclusions and perspectives

The research on tRFs is gaining momentum over the past decade and tRNA-derived fragments have been proved to be involved in various biological functions including regulation of gene expression, translation, and epigenetic regulation. The roles of specific tRFs in several types of cancers and other metabolic disorders are being unraveled. Parental germ cell tRFs have also been shown to influence metabolic disorders in offspring. This opens the doors for additional treatments involving the regulation of the biogenesis of tRFs and their targets and the use of tRFs as potential biomarkers for disease onset. Of particular interest is the evidence of cross-kingdom communication involving tRFs. Detailed studies on such interactions between various infectious agents and their hosts will further help in targeted treatments.

In plants, tRFs have been implicated in biotic and abiotic stress tolerance and transgenerational memory of stress. tRFs acting as signal molecules for interspecies communication deserves specific attention. Altering expression levels of tRFs and their targets can modify traits in target species. In many species, the population of tRFs was found to show differences between tissues and developmental stages pointing towards additional roles for these small non-coding RNAs in the growth and development of organisms.

New sequencing technologies and bioinformatics tools are being used to bypass the complications in tRFs library construction and expression profiling and further research will unravel additional layers of tRFs biogenesis and functions.
